# Ubiquitination, Biotech Startups, and the Future of TRIM Family Proteins: A TRIM-Endous Opportunity

**DOI:** 10.3390/cells10051015

**Published:** 2021-04-25

**Authors:** Utsa Bhaduri, Giuseppe Merla

**Affiliations:** 1 PhD Programme in Molecular Biomedicine, Department of Life Sciences, University of Trieste, Via E. Weiss 2, 34128 Trieste, Italy; u.bhaduri@operapadrepio.it or; 2 Laboratory of Regulatory and Functional Genomics, Fondazione IRCCS Casa Sollievo della Sofferenza, Viale Cappuccini 1, 71013 San Giovanni Rotondo, Italy; 3 Department of Molecular Medicine and Medical Biotechnology, University of Naples Federico II, Via S. Pansini 5, 80131 Naples, Italy

**Keywords:** ubiquitination, E3 ligases, TRIM family, startups, drug discovery

## Abstract

Ubiquitination is a post-translational modification that has pivotal roles in protein degradation and diversified cellular processes, and for more than two decades it has been a subject of interest in the biotech or biopharmaceutical industry. Tripartite motif (TRIM) family proteins are known to have proven E3 ubiquitin ligase activities and are involved in a multitude of cellular and physiological events and pathophysiological conditions ranging from cancers to rare genetic disorders. Although in recent years many kinds of E3 ubiquitin ligases have emerged as the preferred choices of big pharma and biotech startups in the context of protein degradation and disease biology, from a surface overview it appears that TRIM E3 ubiquitin ligases are not very well recognized yet in the realm of drug discovery. This article will review some of the blockbuster scientific discoveries and technological innovations from the world of ubiquitination and E3 ubiquitin ligases that have impacted the biopharma community, from biotech colossuses to startups, and will attempt to evaluate the future of TRIM family proteins in the province of E3 ubiquitin ligase-based drug discovery.

## 1. At the Juncture of Ubiquitination, E3 Ligases, and TRIM Family Proteins: An Introductory Anecdote

Ubiquitin [1], originally reported in 1975 as ‘ubiquitous immunopoietic polypeptide’ (UBIP) [2], is a highly conserved protein of 76 amino acids expressed in all eukaryotic cells [2]. Ubiquitination (also known as ubiquitylation or ubiquitinylation), an ATP-dependent form of protein degradation distinct from lysosomal protein breakdown, [3] was identified later in 1983 as a cascade enzymatic reaction involving three steps. First, ubiquitin is activated by an E1 activating enzyme involving a thioester linkage with the catalytic cysteine. Second, ubiquitin is transferred, through a trans-esterification reaction, to an E2 conjugating enzyme. In the third step, ubiquitin is transferred from the charged E2 to the target substrate by an E3 ligase enzyme, which aids isopeptide bond formation between the C-terminal glycine of ubiquitin and the substrate lysine residue [4]. Finally, this covalent attachment of ubiquitin directs the target substrate towards 26S proteasome-mediated degradation [5].

The central theme of ubiquitination, and ubiquitin–proteasome system (UPS), is a unique and pivotal regulatory mechanism that controls the global protein levels of eukaryotic cells. It is also significantly involved in a multitude of non-proteasomal cellular and molecular processes like DNA damage response, cell cycle control, nuclear translocation, post-Golgi trafficking, endocytosis, and innate immunity [6]. Since the discovery of UPS, it has progressively become a potential subject of interest in the biopharmaceutical industry, particularly following developments in our understanding of the dysregulation of the ubiquitination-proteasome pathway that can significantly contribute to a large number of diseases, including cancers and neurodegenerative disorders [7]. The therapeutic capacity of targeting proteasome-mediated degradation was successfully demonstrated in 2003 with the FDA approval of proteasome inhibitor bortezomib (Velcade^®^) for the treatment of relapsed or refractory multiple myeloma [8]. However, a paradigm shift in ubiquitin-based drug discovery came a little earlier, in 2001, with the concept of PROteolysis TArgeting Chimeras (PROTACs), where SCFβ-TRCP E3 ubiquitin ligase was used to induce the targeted protein degradation (TPD) of methionine aminopeptidase-2 (MetAp-2) [9,10]. This replacement of traditional ‘inhibition’ with ‘inducing degradation’ using E3 ligases provided fertile ground for a new age biopharma slogan, ‘drugging the undruggable’ [11], and ushered in a new era of ubiquitin-inspired startups and technological innovations. Degradation has now become the preferred choice over inhibition, and E3 ligases, the largest and most heterogeneous family of proteins in the ubiquitin pathway, with ~700 members (representing ~5% of the human genome), have come to the forefront of drug discovery [11,12].

**Figure 1:**
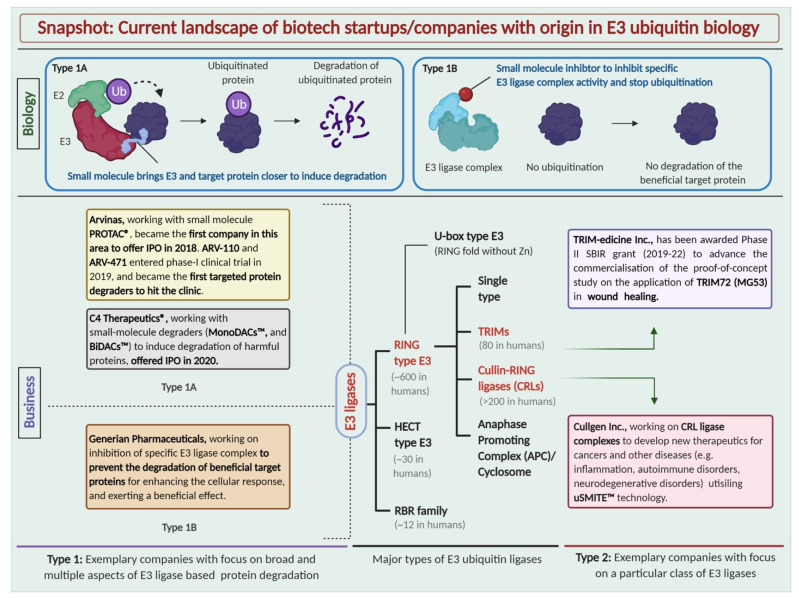
Types of E3 ubiquitin ligases and the current landscape of biotech startups with their origin in E3 ubiquitin biology. Schematic representation of exemplary startups working with different approaches in the space of E3 ubiquitin drug discovery.

Depending on the presence of characteristic domains and the mechanism of ubiquitin transfer to the substrate protein, E3 ligases are classified into three major types: RING E3s, HECT E3s, and RBR E3s (Figure 1) [13]. Tripartite motif containing proteins, commonly known as TRIMs, are classified as RING E3s, mainly due to the presence of RING domains in their structures, and have recently emerged as important players in carcinogenesis, DNA repair, and metastasis. Some TRIMs have already been established as critical regulators of autophagy, innate immunity, intracellular signaling, and transcription [14]. Recently, we reported that TRIM8 is a ‘molecule of duality’ (MoD) because of its dual role as both a tumor suppressor and an oncogenic molecule [15]. Over the past two decades, during which the basic biological roles and characteristics of TRIM E3 ligases have begun to be elucidated, several studies have reported potential therapeutic applications of multiple other RING and HECT E3 ligases (excluding TRIMs) across a wide variety of pathophysiological conditions [16,17]. In particular, the last decade of drug discovery (i.e., 2011–2020) has shown an increase in the formation of new biotech start-ups and the development of novel technologies in E3 ligase-mediated protein degradation. And simultaneously, the biotech market has eye-witnessed the successful 2018 initial public offering (IPO) of Arvinas, which became the first unicorn company (a privately held startup that has reached a valuation of over USD 1 billion) in the area of TPD. Meanwhile, in 2019, for the first time, PROTAC degraders ARV-110 and ARV-471 entered phase 1 clinical trials for the treatment of prostate cancer and breast cancer, respectively [18].

In this review, in addition to accumulating information on recent developments in the science and business of E3 ubiquitin ligases, the growing importance of TRIM E3 ligase proteins in multiple disorders is reviewed, and an attempt is made to evaluate the important role that TRIMs could potentially play in the next decade (which we term the ‘third decade’, i.e., 2021–2030) of E3 ubiquitin drug discovery.

## 2. E3 Ligases, an Inflection Point in the Biotech Industry, and the Rise of TRIM Family Proteins

### 2.1. At the Crossroads: E3 Ligases and Biomedicine

The biotechnology and biopharmaceutical industries have grown rapidly over the past decade. As of 2017, four sub sectors of the biopharmaceutical industry—‘pharmaceuticals’, ‘biotechnology therapeutics’, ‘small molecule therapeutics’, and ‘gene therapy’ have found themselves among the top thirty most attractive industry fields to investors [19]. Interestingly, when the biotech industry was approaching the beginning of the human genomics era following the publication of *Initial sequencing and analysis of the human genome* on 15 February 2001, the FDA approval of tyrosine kinase inhibitor Gleevec^®^ (imatinib) in the same year also helped lay the foundations for a new age of biomedicine (medicine developed through research in molecular biology) and establish the high market potential of kinase inhibitors, which accounted for $40.41 billion in 2019 and is projected to reach $64.90 billion by 2027 [20]. Even though bortezomib (Velcade^®^), the first drug targeting the ubiquitin–proteasome pathway, was approved by the FDA in 2003 [8], the global market value of UPS-based drugs did not see the same unprecedented market growth that kinase inhibitors did in the first decade of E3 ubiquitin drug discovery (2001–2010), primarily due to the lack of biological understanding of the very large E3 ubiquitin ligase family. The scenario changed, however, in the second decade of E3 ubiquitin drug discovery (2011–2020), due to the development of many novel technologies, the growing interest that biotechnology-focused venture capital (VC) firms showed in backing innovative startups working in E3 ubiquitin drug discovery, and the progress made in our biological understanding of many E3 ligases. Following two successful IPOs of E3 ubiquitin drug discovery startups, Arvinas in 2018 [21] and C4 Therapeutics in 2020 [22], it is now widely believed that targeting the ubiquitin-proteasome pathway is revolutionary, and possess the same market growth potential that kinase inhibitors once exhibited in the coming decade, i.e., the third decade of E3 ubiquitin drug discovery (2021–2030).

### 2.2. E3 Ligases and Targeted Protein Degradation: Drugging the Undruggable

**Figure 2:**
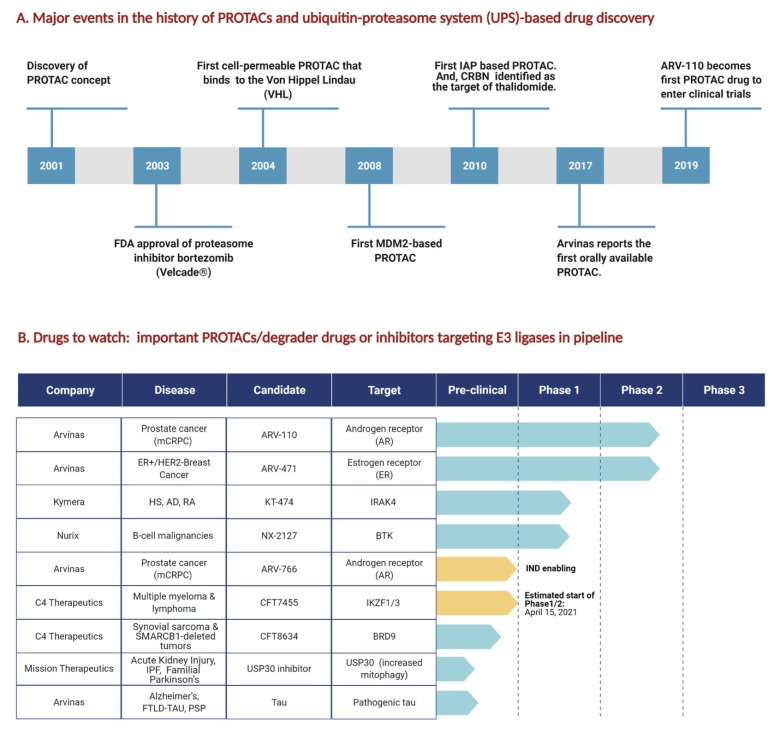
Major events in the history of PROTAC and UPS-based drug discovery and highlights of important PROTACS/E3 ubiquitin ligase-based drugs in the pipeline. (**A**) Timeline showing developments in the history of drug discovery in UPS with focus on PROTACs. (**B**) Important PROTACs/degrader drugs or inhibitors targeting E3 ligases that are currently in the clinical pipeline at the biotech startups that were founded in the second decade of E3 ubiquitin drug discovery (2011–2020). IND: investigational new drug.

Historically, the classical pharmaceutical drug development approach has been based on the identification of drug targets with an active site that can accommodate a small molecule. Therefore, traditional pharmaceutical drug development was primarily limited to the identification of active site-containing targets and the discovery of small molecule inhibitors [10]. Although successful in many cases, a major limitation of this method is that these small molecule inhibitor drugs necessitate a strong binding capacity to the target protein, mostly to its active site. Moreover, as Pettersson et al. noted, this ‘occupancy-driven pharmacology’ is not applicable to all biological targets, especially those that lack enzymatic activities [10]. These limitations of the traditional drug discovery approach meant that ~80% of the proteome was ‘undruggable’. The discovery of PROTACs in 2001 signaled a major paradigm shift in the drug discovery approach, where TPD became the choice over classical inhibition. PROTACs are bifunctional, two-headed, small molecules connected by an optimal linker, having one head (the protein of interest ligand) that selectively binds a target protein and a second head (the E3 ubiquitin ligase recruiting ligand) that binds to an E3 ubiquitin ligase, and thus brings the E3 ligase into the proximity of a specific disease-causing target protein so that it can be tagged with ubiquitin and subsequently degraded by the 26S proteasome. This new method of drug discovery brought with it hope that many disease-causing proteins, which were previously thought to be undruggable under the traditional approach, could be targeted, as PROTAC protein degraders have the capacity to function even by binding weakly to a target protein [9,10]. Over the past decade, based on the further development of this protein degradation technology in UPS drug discovery (Figure 2A), combined with the progress in our understanding of the function of several E3 ligases, many biotechnology startups have built business innovation models around the clinical pipelines of various diseases, including but not limited to cancers and neural disorders (Figure 2B).

### 2.3. The Advent of TRIM Family Proteins in the E3 Ligase Family

In 2001, TRIM family proteins (TRIMs), also known as RBCC proteins, were characterized by the presence of tripartite motif (TRIM) or three domains: a RING-finger domain (R), a B-box domain (B), and a Coiled-Coil domain (CC) (although not all TRIM proteins have a RING domain) [23]. For instance, eight TRIMs in humans are RING-less [14]. TRIMs/RBCC proteins were later included as a novel class of RING E3 ligases in 2003, as many of the TRIM proteins were reported to be involved in the process of ubiquitination and shown to possess E3 ligase activity [24]. In humans, so far, there are 80 annotated TRIM proteins, many of which are now known to be involved in different kinds of ubiquitination processes like K48, K63, and K6-linked ubiquitination. Several studies have reported that many of the TRIM family proteins play a pivotal role in the progression of different cancers [25]. Interestingly, our growing understanding of them suggests that TRIMs are also significantly involved in several non-cancerous diseases like rare genetic disorders, neural and neurodegenerative disorders, and cardiac disorders (Table 1), making TRIMs a considerable choice for carrying out further studies in the third decade of E3 ubiquitin drug discovery [26].

## 3. TRIMs and Cancers: Involvement with Multiple Approaches

Many of the TRIM family proteins are now known to be involved in key oncogenic processes like cell proliferation, apoptosis, and transcriptional regulatory circuitries [25]. Some TRIM proteins are known to be upregulated in cancers whereas some are downregulated [27]. TRIM29 is highly overexpressed in many cancers like lung cancer, bladder cancer, colon cancer, ovarian cancer, and multiple myeloma, whereas TRIM19 is associated with t(15;17)(q24;q21) translocation, which is a key driving agent for acute promyelocytic leukaemia (APL) [25]. TRIM27 has recently been predicted as a prognostic marker of melanoma [28]. Below, we have described the involvement of major TRIMs in cancers through multiple approaches.

### 3.1. Epigenetic Approach

TRIM24, also called as Transcription Intermediary Factor 1 alpha (TIF1α), is involved in the regulation of TP53 stability and functions as a human oncogene in multiple cancers like cervical, prostate, head and neck, and glioma when it is aberrantly overexpressed [29,30]. TRIM24, TRIM28, TRIM33, and TRIM66 form the transcriptional intermediary factor 1 (TIF1) family of chromatin-binding proteins that are involved in DNA damage response (DDR) and show significant involvement in different cancer types [30]. Interestingly, TRIM24, TRIM28, and TRIM33 come under Class VI of the TRIM family of proteins, which share the common structure of RBCC motif and a C-terminal PHD and bromodomain. Even though TRIM66 does not have the RING domain, it maintains commonality by sharing a B-box, Coiled-Coil, and C-terminal PHD-bromodomain. It is well known that PHD and Bromodomain are very important in chromatin biology and transcriptional regulation, and they can function as versatile “readers” of histones and histone modifications [31]. Earlier studies have shown that TRIM24 interacts directly with euchromatin through its C-terminal PHD-bromodomain [32,33], and TRIM24 PHD-bromodomain can function as a single functional unit for combinatorial recognition of post-translational modifications such as H3K27ac (histone H3 acetylated at lysine 23) and H3K4me0 (unmodified H3K4) [34]. Furthermore, TRIM24 PHD-bromodomain can also interact with histone H3 peptides methylated at K9 (H3K9me3), a well-known mark associated with transcriptional repression. Proteomic study of H3K9me3-associated protein complexes has discovered TRIM24 along with TRIM28 and TRIM33 in mouse brain, testis, and kidney [29,35]. Overall, the functional similarity of these TIF1-TRIMs in the context of chromatin biology is very clear due to the presence of PHD-bromodomain. Most importantly, an oncogenic TRIM family protein can also protect another oncogenic member of the family, and thus facilitate the progression of cancer. For instance, recent a study has shown that TRIM28 protects TRIM24 from its ubiquitination and degradation by E3 ubiquitin ligase adaptor SPOP and fosters prostate cancer progression. TRIM28 has now been established as a positive regulator of TRIM24 stability through protein-protein interactions [36].

### 3.2. Stemness Approach

Many of the TRIM family proteins are known as critical regulators of stemness and stem-cell renewal. TRIM6, TRIM11, TRIM14, TRIM19, TRIM24, TRIM25, TRIM27, TRIM28, and TRIM71 act as positive stem-cell regulators, whereas TRIM16, TRIM21, and TRIM32 act as negative regulators of cell stemness [37]. Interestingly, TRIM8 can function as both a positive and a negative regulator of cell stemness by activating STAT3 signaling and by inhibiting translocation of STAT3 into the nucleus, respectively [37]. Overexpression of TRIM8 leads to the higher expression of p-STAT3, c-MYC, SOX2, NESTIN, and CD133, and enhances self-renewal of glioblastoma stem cells. TRIM8 regulates stemness in glioblastoma by activating STAT3 signaling and inhibiting the expression of PIAS3 [38,39]. Alongside TRIM8, TRIM28 is also essential in maintaining the stem cell characteristics of cancer cells. Downregulation of TRIM28 reduces the ability of cancer stem cells to self-renew, which significantly reduces tumor growth [40]. TRIM32, TRIM6, TRIM19, and TRIM28 interact with key stem cell transcription factors (SCTFs) like c-MYC, Oct4, and SOX2. TRIM32, particularly, has the capacity to ubiquitinate c-MYC, and, along with deubiquitinase USP7, it balances the level of c-Myc ubiquitination [41]. Furthermore, TRIM19 (PML) is reported as the essential regulator of stem cell pluripotency and somatic cell reprogramming, and associates with Oct-3/4, STAT3, c-MYC, and NR0B1 to maintain the naive pluripotent state [42].

### 3.3. Signaling Pathways Approach

TRIM proteins are involved in the regulation of highly important cancer related signaling pathways like TP53, JAK/STAT, Hedgehog, Wnt/beta-catenin, Notch, PI3K/Akt, TGF-β, and NF-κB. Oncogenic TRIMs, like TRIM11, TRIM21, TRIM23, TRIM24, TRIM25, TRIM28, TRIM29, TRIM31, TRIM32, TRIM39, TRIM59, and TRIM66, directly or indirectly inhibit the activity of tumor suppressor protein p53, whereas other TRIMs, such as TRIM3, TRIM13, TRIM19, and TRIM67, can exercise their anti-cancer capacity by inducing the activity of p53 [27]. In contrast, TRIM8 is an exception, which can function as both a tumor suppressor and an oncogenic molecule. p53 lies at the center of TRIM8’s tumor suppressor activity, whereas TRIM8, along with many other oncogenic TRIMs, functions as a pro-cancerous molecule through the regulation of the NF-κB pathway [15]. TRIM66 is reported to promote the progression of prostate carcinoma through the JAK/STAT pathway [43]. TRIM29 and TRIM58 modulate the Wnt/β-catenin signaling pathway, and TRIM14, TRIM27, TRIM44, and TRIM59 are known to be involved in the activation of the PI3K/Akt pathway in different tumours [27].

### 3.4. RNA Binding Approach

RNA-binding proteins (RBPs) are attracting global attention due to the diversified roles of functional crosstalk between proteins and RNAs [44,45]. Class VII of TRIM family proteins having NHL domains (TRIM2, TRIM3, TRIM32, and TRIM71) are emerging as a unique class of RNA binding ubiquitin ligases (RBULs) [46], alongside a few other TRIMs with a C-terminal PRY/SPRY domain (TRIM25, TRIM26 and TRIM65), a bromodomain (TRIM28 and TRIM33), and RING-less TRIMs like TRIM44 [47]. TRIM-NHL proteins are conserved from lower organisms like Drosophila and *C. elegans* to mammals, and are known to regulate a large variety of mRNAs [46]. For instance, the NHL-domain of brain tumors (Brat), an orthologue of human TRIM3 in Drosophila, is reported to bind with the 3′UTR of hunchback (hb) mRNA through the involvement of two RBPs, Nanos and Pumilio, during oogenesis [47,48,49]. Several earlier studies have confirmed the RNA-binding capacity of the orthologues of human TRIM-NHL proteins in Drosophila and *C. elegans* [50]. Moreover, miRNA regulation activity of TRIM-NHL members has also been well studied in the worm and mice. NHL-2, an orthologue of human TRIM32 in the worm, positively regulates miRNA activity and is essential for let-7 and lsy-6 miRNA functions [51]. Interestingly, TRIM-NHL proteins are now known to play an important role in cancers and tumorigenic processes. Liu et al. reported that *TRIM32* is a direct target gene of TP53, and that TRIM32, in turn, promotes tumorigenesis as a negative regulator of TP53 by degrading the tumor suppressor protein through ubiquitination [52]. TRIM3 has been characterized as a tumor suppressor, and it regulates the asymmetric cell division in glioblastoma stem cells [53]. TRIM71 is also reported to act as a tumor suppressor due to its involvement in the post-transcriptional modulation of Lin28B-let-7-HMGA2 (high-mobility group AT-hook 2) signaling in tumorigenesis [54].

## 4. E3 Ligases in the Post ‘Genomic Bubble’ Burst Scenario: Industrial and Entrepreneurial Landscape

**Figure 3:**
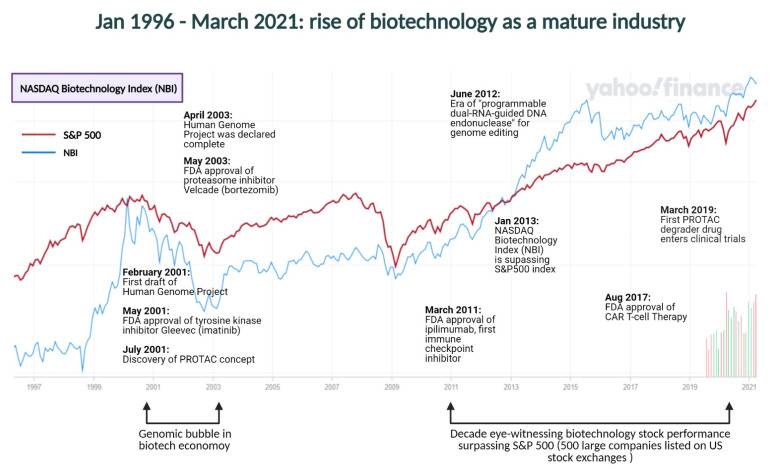
NASDAQ Biotechnology Index (NBI) and S&P 500 stock exchange data on a logarithmic scale. As of January 2013, the NASDAQ Biotechnology Index (blue) has surpassed the S&P 500 stocks (red), maintaining record growth. Some of the historic events in the life sciences that have influenced the biotechnology industry have also been marked on the timeline. Data source: NASDAQ Biotechnology Index. Credit: Yahoo Finance.

During the early 2000s’ ‘genomic bubble’, many biotech start-ups went public, raised their valuation, and then failed [55,56]. This created the initial impression of biotech hype, which brought about the realization of the limitations of genomics. As Stanley Fields famously stated, “the future belongs to proteomics” [57]. However, the overall growth of the biotech industry changed speedily after 2010 (Figure 3) with multiple positive factors like the success and market growth of kinase inhibitors, FDA approvals of immune checkpoint inhibitors, large-scale investment attracted by cell and gene therapy companies, rapid involvement of artificial intelligence in drug discovery, and the long-term scientific impact of the Human Genome Project. NASDAQ Biotechnology Index (NBI) data clearly show, as of 2017, in tandem with ‘big pharma’ (pharmaceuticals), biotech has emerged as a mature industry with a good number of innovative, science-driven companies [58]. Between 31 December 2012 and 22 June 2017, the NASDAQ Biotechnology Index has outperformed both the S&P 500 Index (cumulative return of 71%) and the S&P 500 Healthcare Index (cumulative return of 101%), with a cumulative return of 134% [58]. In the last three years, from December 2017 until December 2020, the NASDAQ Biotechnology Total Return Index (XNBI) stands at 44.15%, while it stands at 409.78% in the long-term observation between December 2010 and December 2020. Having this positive vibe as the backdrop to the biotech market, we identify many innovative biotech startups with a strong focus on E3 ligases and targeted protein degradation that were founded between 2011 and 2020, i.e., the second decade of E3 ubiquitin drug discovery (Table 2). Towards the end of this second decade, we also see that four innovative startup companies, Arvinas, Neurix Therapeutics, C4 Therapeutics, and Kymera Therapeutics, offered their IPOs and went public. Most importantly, in 2019, Arvinas brought two PROTAC degrader drugs, for the first time in the history of targeted protein degradation, into the clinic [18]. In Q1 of 2021, C4 therapeutics announced the FDA approval of their investigational new drug (IND) application for Phase 1/2 clinical trial of CFT7455 [59].

Although there are eight known human E1 or E1-like enzymes (two major ones are UBE1 and UBA6) [60] alongside ~40 E2 conjugating enzymes [61], targeting E3 ligases has always been the preferred choice over E1 activating and E2 conjugating enzymes because of their very large family size, distinct catalytic mechanisms, and the possibility of bringing better target specificity to drug discovery [11]. In the realm of UPS-based drug discovery, there are two major approaches that utilize the biological capacity of E3 ligases. First, a disease-causing E3 ligase can be targeted with small molecule inhibitors. For instance, the E3 ligase cereblon (CRBN) has been used for the targeted treatment of different diseases by FDA-approved chemical inhibitors like thalidomide (multiple myeloma, graft-versus-host disease), lenalidomide (myelodysplastic syndrome, multiple myeloma), and pomalidomide (multiple myeloma) [62]. There are also a few other E3 ligases, like MDM2, Skp2, Cdc4, and βTrCP, for which drug compounds are being developed [63]. Second, an E3 ligase can be brought closer to a target substrate with a small molecule degrader drug like PROTAC to control the protein level of the target substrate. Even though PROTAC or PROTAC-like small molecule degrader drug development has become very popular in the last few years, the number of E3 ligases that are currently being used for targeted protein degradation remains very small. Some of the most prominent E3 ligases that are being explored for small molecule degrader drug development are CRBN, von Hippel–Lindau (VHL), and inhibitors of apoptosis proteins (IAPs) [64]. Particularly, CRBN and VHL have emerged as the two most preferred E3 ligases for PROTAC degrader development for their many favorable biophysical and structural properties [64]. In the last decade, our biological understanding about TRIMs and their involvement in multiple diseases has enormously expanded. Due to the availability of structural information, TRIM24 was first targeted with a potent acetyl-lysine mimetic benzimidazolones TRIM24 bromodomain inhibitor [65]. dTRIM24, a bifunctional PROTAC degrader of TRIM24, was designed later for the selective degradation of TRIM24 facilitated by the recruitment of VHL E3 ligase [66]. There has been, however, no attempt reported so far that involves the use TRIM24 as a successful E3 ligase for the targeted degradation of selected substrates. Recently, ‘TRIM-away’ technology has been developed for the rapid degradation of endogenous proteins based on ubiquitin ligase and Fc receptor, TRIM21. In this method, antibodies are introduced to a target protein, and then TRIM21 is recruited by the antibody bound target protein followed by the proteasome-mediated degradation of the target protein and antibody-bound TRIM21 complex [67]. Although it is yet to be explored, there is immense opportunity for developing new drugs using TRIMs in many other ways like targeting the substrate binding domains (e.g., PRY/SPRY, NHL), exploiting the homo/hetero-typic interactions among TRIMs, and the possibility of interfering in the catalytic activation paths of TRIMs’ E3 ligase activity [68,69,70]. At the same time, there are also some major reasons that have slowed the pace of TRIM family proteins to enter the pharmaceutical industry. In the coming years, we believe that with the availability of the structures of more TRIMs (as most of them are currently unsolved) and the biological elucidation of more defined target substrates of TRIMs, the path into the pharmaceutical industry for this unique class of RING E3 ligases will be paved smoothly.

## 5. The Future of TRIMs and the TRIM-Endous Opportunity: Concluding Remark

Overall, we can say that E3 ubiquitin ligase-based drug discovery research is moving towards a promising future in its third decade (2021–2030), and its sphere of influence is progressively growing due to its collaborative business model with big pharma and backing from biotechnology focused venture capital firms at different stages of investment. As there are now many PROTACs and E3 ubiquitin ligase-based small molecule drugs in the pipeline from multiple biotech startups or companies at different pre-clinical and clinical stages, it will be a crucial decade for UPS and E3 ligase-based drug discovery, and the coming years will reveal whether it meets the expectations set by the earlier success of biotech blockbusters like kinase inhibitors. We strongly believe, in this third decade, a “TRIM-endous” (tremendous opportunity for TRIMs) scientific and entrepreneurial initiative will be required to elucidate the more detailed biological function of TRIMs and to design PROTACs/degrader drugs against TRIMs or to utilize TRIMs as the E3 ligase for targeted protein degradation. For instance, although TRIM19 (PML) and TRIM33 three-dimensional crystal structures are available with bound ligands or partner substrate proteins, they have yet to be taken forward for targeted protein degradation or to design degrader drugs. Moreover, what the experimental and clinical suitability of TRIMs for use as the E3 ligase in a targeted protein degradation for drug development is remains largely unknown, along with the structural information about most TRIMs. Furthermore, due to the growing evidence for TRIMs and E3 ligases’ capacity to function as RBP or RBUL, we also predict that this third decade has the potential to emerge as a melting pot of two of the hottest drug development approaches of our time—RNA therapeutics and TPD.

## Figures and Tables

**Table 1:** Role beyond cancers: involvement of TRIMs in multiple non-cancerous disorders.

Therapeutic Area	Disease	TRIM	PMID
Cardiovascular and metabolic diseases	Hypertrophic cardiomyopathy (HCM)	TRIM63	PMID: 32451364
	Cardiac hypertrophy	TRIM8	PMID: 27956576
		TRIM32	PMID: 26884348
	Cardiomyopathy	TRIM76 (CMYA5)	PMID: 18344630
	Diabetic cardiomyopathy, cardiac I/R Injury	TRIM72 (MG53)	PMID: 28432201
Neurological, neurodegenerative, and neuropsychiatric diseases	Schizophrenia	TRIM76 (CMYA5)	PMID: 20838396
		TRIM1 (MID2)	PMID: 24115387
		TRIM19 (PML)	PMID: 21822266
	Alzheimer’s disease	TRIM20	PMID: 17090974
	Parkinson’s disease	TRIM8	PMID: 32463919
		TRIM32	PMID: 28508149
		TRIM10	PMID: 28586827
	Epilepsy	TRIM8	PMID: 30244534
		TRIM27	PMID: 33385414
	Multiple sclerosis (MS)	TRIM5	PMID: 21311761
		TRIM10	PMID: 28586827
	HTLV-1-Associated Myelopathy (HAM/TSP)	TRIM22	PMID: 29872426
		TRIM5	PMID: 29872426
Developmental diseases	Limb-girdle muscular dystrophy 2H	TRIM32	PMID: 21496629
	Mulibrey nanism (MUL)	TRIM37	PMID: 27044324
	Williams-Beuren syndrome (WBS)	TRIM50	PMID: 18398435
Muscle weakness	Thyrotoxic periodic paralysis (TPP)	TRIM2	PMID: 33105104
Autoimmune disease	Celiac disease	TRIM10	PMID: 28586827

**Table 2:** Major startups founded in the second decade of E3 ubiquitin drug discovery between 2011 and 2020.

Companies	Founded	IPO	Bedrock Science/Technology	Broad Therapeutic Areas
Mission Therapeutics	2011	No	Designing inhibitors to target specific disease-associated deubiquitinating enzymes (DUBs).	Acute Kidney Injury, Idiopathic Pulmonary Fibrosis, Familial Parkinson’s Disease
Nurix Therapeutics	2012	Yes (2020)	Utilising DELigase™ drug discovery platform to harness an E3 ligase to ubiquitinate disease-associated proteins and also to discover novel E3 ligase inhibitors for increasing the target proteins.	Cancers, and Autoimmune Diseases
Arvinas	2013	Yes (2018)	PROTAC^®^ discovery engine and “Arvinas Rules” (alternative to “Rule of 5”) to create PROTAC degrader drugs.	Cancers, and Neural Disorders (Alzheimer’s, Parkinson’s, Huntington’s)
C4 Therapeutics	2016	Yes (2020)	C4T TORPEDO™ platform (Target ORiented ProtEin Degrader Optimizer) for catalysing discovery of degrader drugs. Working with two different types of degraders MonoDACs™ and BiDACs™.	Cancers
Kymera Therapeutics	2016	Yes (2020)	Pegasus platform for designing degrader drugs.	Cancers, and Immune Diseases
Captor Therapeutics	2017	Expected soon (2021)	Optigrade™ technolgy platform for targeted protein degradation (TPD) platform.	Cancers, and Autoimmune Diseases
Amphista Therapeutics	2017	No	Working with bifunctional molecules ‘Amphistas’ for targeted protein degradation.	Cancers, and Neural Disorders
Cullgen	2018	No	Working with uSMITE™ technology to explore cullin-RING E3 ubiquitin ligases (CRLs) for targeted protein degradation.	Cancers, and Immune Diseases
Polyprox Therapeutics	2018	No	Polyproxin^®^ platform to exploit protein-protein interactions for harnessing proteostasis networks and targeted protein degradation.	Cancers
Generian Pharmaceuticals	2019	No	Exploiting E3 ligase biology to modulate the activity of network regulators and therapeutic targets TFEB and AMPK.	Autophagy and Lysosomal Storage Diseases, and Metabolic and Mitochondrial Function Disorders
Monte Rosa Therapeutics	2020	No	Exploring the capacity of glycine loop degrons for targeted protein degradation of disease-causing proteins like transcription factors and adaptor proteins using E3 ligases.	Cancers, and Genomically-Defined Diseases

## Abbreviations

Tripartite motif	TRIM
ubiquitin–proteasome system	UPS
PROteolysis TArgeting Chimeras	PROTACs
targeted protein degradation	TPD
initial public offering	IPO
venture capital	VC
RNA-binding proteins	RBPs
RNA Binding Ubiquitin Ligases	RBULs
500 large companies listed on the US stock exchanges	S&P 500
